# Impact of thrombus aspiration during ST-Elevation Myocardial Infarction: a six month composite endpoint and risk of stroke analyses of the TASTE trial

**DOI:** 10.1186/s12872-016-0238-y

**Published:** 2016-04-01

**Authors:** Göran K Olivecrona, Bo Lagerqvist, Ole Fröbert, Thórarinn Gudnason, Michael Maeng, Truls Råmunddal, Jan Haupt, Thomas Kellerth, Jason Stewart, Giovanna Sarno, Jens Jensen, Ollie Östlund, Stefan K James

**Affiliations:** Department of Cardiology, Skane University Hospital, Lund University, Lund, 221 85 Sweden; Department of Medical Sciences, Cardiology and Uppsala Clinical Research Center, Uppsala University, Uppsala, Sweden; Department of Cardiology, Örebro University, Faculty of Health, Örebro, Sweden; Department of Cardiology, Landspitali University Hospital, Reykjavik, Iceland; Department of Cardiology, Aarhus University Hospital, Skejby, Aarhus, Denmark; Department of Cardiology, Sahlgrenska University Hospital, Gothenburg, Sweden; PCI Unit, Sunderby Hospital, Sunderby, Sweden; Department of Cardiology, Skaraborgs Hospital, Skövde, Sweden; Department of Cardiology, Karolinska Institutet, Stockholm and Sundsvall Hospital, Sundsvall, Sweden

**Keywords:** Myocardial infarction, STEMI, PCI, Thrombus aspiration

## Abstract

**Background:**

Routine thrombus aspiration during primary percutaneous coronary intervention (PCI) in ST-elevation myocardial infarction (STEMI) did not reduce the primary composite endpoint in the “A Randomised Trial of Routine Aspiration ThrOmbecTomy With PCI Versus PCI ALone in Patients With STEMI Undergoing Primary PCI” (TOTAL) trial. We aimed to analyse a similar endpoint in “The Thrombus Aspiration in ST-Elevation myocardial infarction in Scandinavia” (TASTE) trial up to 180 days.

**Methods:**

In TASTE, 7244 patients with STEMI were randomised to thrombus aspiration followed by PCI or to PCI alone. We analysed the quadruple composite endpoint of cardiovascular death, cardiogenic shock, rehospitalisation for myocardial infarction, or new hospitalisation for heart failure. Furthermore, an extended net-benefit composite endpoint including stent thrombosis, target vessel revascularization or stroke within 180 days was analysed.

**Results:**

The primary quadruple composite endpoint occurred in 8.7 % (316 of 3621) in the thrombus aspiration group compared to 9.3 % (338 of 3623) in the PCI alone group (hazard ratio (HR), 0.93; 95 % confidence interval (CI); 0.80 - 1.09, *P* = 0.36) and the extended net-benefit composite endpoint in 12.0 % (436) vs. 13.2 % (479) (HR, 0.90; 95 % CI; 0.79 - 1.03, *P* = 0.12). Stroke within 30 days occurred in 0.7 % (27) vs. 0.7 % (24) (HR, 0.89; 95 % CI; 0.51–1.54, *P* = 0.68).

**Conclusions:**

A large and an extended composite endpoint analysis from the TASTE trial did not demonstrate any clinical benefit of routine thrombus aspiration during PCI in patients with STEMI. There was no evidence of an increased risk of stroke with thrombus aspiration.

## Background

Thrombus aspiration as adjunctive therapy to primary percutaneous coronary intervention (PCI) has been a much debated subject with conflicting results from both randomised clinical trials and retrospective analysis from registry databases [1–5]. The two largest randomised trials, “The Thrombus Aspiration in ST-Elevation myocardial infarction in Scandinavia” (TASTE, 7244 patients) trial and the recently published “A Randomised Trial of Routine Aspiration ThrOmbecTomy With PCI Versus PCI ALone in Patients With STEMI Undergoing Primary PCI” (TOTAL, 10 732 patients) trial were both negative regarding their primary endpoints; death at 30 days in TASTE and the composite endpoint of cardiovascular (CV) death, recurrent myocardial infarction (MI), cardiogenic shock or new or worsening New York Heart Association (NYHA) class IV heart failure (HF) within 180 days in TOTAL [1, 5]. Together, the TASTE and TOTAL trials dwarf all previous randomised trials of thrombus aspiration in number of patients randomised, but criticism has been raised towards the TASTE trial regarding an underpowered study with inclusion of a low risk population, lack of evaluation of stroke rates and occurrence of HF post index hospitalisation as well as no adjudication of events [6]. However, the TASTE trial was unique because it was a prospective simple registry based randomised clinical trial (R-RCT) with randomisation and events solely performed and collected through national health and death registries. We now wanted to analyse the TASTE cohort of patients, using similar endpoints collected in the TOTAL trial, captured up to 180 days for a composite endpoint of CV death, rehospitalisation for new MI, cardiogenic shock and rehospitalisation for HF and also collect data for the key safety outcome of stroke up to 30 and 180 days which has come into question following publication of the TOTAL trial.

## Methods

### Study designs

The TASTE trial, ClinicalTrials.Gov Identifier NCT01093404, was approved for Sweden (all Swedish sites) by the Regional Ethical Committee in Uppsala, for Denmark by the Ethical Committee Region Midt Jylland, Århus, and for Iceland by the ethical committee at Landspítali University Hospital Reykavik. The study complies with the Declaration of Helsinki. Signed informed consent was obtained from all patients.

The design, baseline characteristics and results of the TASTE and TOTAL trials have been published previously [1, 5–8]. Both are multicentre, prospective, randomised clinical open-label trials in patients with ST-elevation myocardial infarction (STEMI) evaluating use of routine thrombus aspiration, before PCI, as compared to PCI alone. Patients were randomised in a 1:1 fashion. The TASTE trial performed identification of patients, randomization, collection of baseline and procedural variables and follow-up from the “Swedish Web-system for Enhancement and Development of Evidence-based care in Heart disease Evaluated According to Recommended Therapies” registry (SWEDEHEART), the “Swedish angiography and angioplasty registry” (SCAAR), and national population registries [9]. The TOTAL trial is a classical randomised clinical trial (RCT) with monitoring of patients and adjudication of events as well as independent core lab analysis of angiographic films.

The primary composite endpoint of TOTAL encompassed data of new or worsening HF (NYHA class IV) and cardiogenic shock which has not previously been reported from the TASTE trial. The primary safety endpoint of stroke up to 30 and 180 days has also not previously been reported from the TASTE trial. Data was therefore captured from the TASTE cohort using the international classification of disease (ICD) code database within the Swedish national database “The National Patient Registry” (NPR) from the Swedish National Health Board (http://www.socialstyrelsen.se/english). Data from the NPR database was then jointly analysed with the TASTE database in SCAAR/SWEDEHEART. The small number of patients from Denmark and Iceland were entered in separately by the investigators.

### Important differences between TASTE and TOTAL

The main difference between the two trials were the primary endpoints, all-cause mortality vs. a composite endpoint. Furthermore, the TASTE trial included patients up to 24 h after onset of symptoms as opposed to 12 h in TOTAL. Also, patients with previous coronary artery bypass grafting (CABG) were excluded from TOTAL. Finally, patients were randomised after the diagnostic coronary angiogram in TASTE whereas patients in TOTAL were randomised before the coronary angiogram although patients who did not undergo PCI for the index STEMI were not included in the TOTAL’s primary analysis.

### Endpoints, outcomes, and definitions

Clinical endpoint parameters were obtained from national health registries as described above in Sweden, Denmark and Iceland. A small number of Danish and Icelandic patients could not be captured for cardiogenic shock and HF (247 Danish for cardiogenic shock and 156 Icelandic for rehospitalisation of HF). Most importantly, no study-specific clinical follow-up was performed. The following endpoints were thus captured; all cause death, CV death, cardiogenic shock, rehospitalisation for new MI, hospitalisation for new HF, stroke, definite stent thrombosis (ST) and target vessel revascularization (TVR) up to 30 and 180 days as well as for the maximum follow up time.

The primary endpoint for the present analysis was the quadruple composite of CV death, cardiogenic shock, rehospitalisation with new MI and hospitalisation for new HF corresponding to the primary outcome of the TOTAL trial. Secondary endpoints include the individual components of the composite in addition to stroke, definite ST, TVR and all cause death. Further evaluated composite endpoints include the net-benefit endpoint comprised of the primary endpoint and stroke and also the extended net-benefit endpoint comprised of the primary endpoint in addition to stroke, definite ST and TVR.

Specifically, all cause death and CV death were captured from the national death registries and national cause of death registries. Cardiovascular deaths were identified inclusively as deaths with the underlying cause ICD code I00-I99 or not recorded, hence only deaths with a recorded non-cardiac underlying cause were excluded. Stroke was captured as ICD code I60-I64, either as primary diagnosis for a new hospitalisation in NPR, or as one of the discharge diagnoses for the index hospitalisation in SWEDEHEART, with strokes during index hospitalisation counted as occurring on the day of randomization.

New hospitalisations with myocardial infarction were captured using the ICD codes I20-22. New hospitalisations for HF were captured as primary diagnosis using ICD code I50 in NPR. Cardiogenic shock was captured for patients with Killip class I-III HF at randomization with a new angio/PCI with Killip class IV in SCAAR or a post-baseline record of cardiogenic chock in SWEDEHEART, including both index and new hospitalisations.

Target lesion revascularization was defined as a new PCI in the same coronary segment as the index procedure or CABG after the index procedure and captured from the SWEDEHEART/SCAAR registry. Stent thrombosis was defined as definite ST and captured from the SWEDEHEART/SCAAR registry.

All endpoints in Denmark and all non-SWEDEHEART based end points in Iceland were collected by the investigators.

### Statistics

The results were analysed according to the intention-to-treat principle, i.e. patients randomised to a given group were followed and assessed irrespective of the treatment. Time to the composite endpoint was presented in a Kaplan-Meier plot. The treatment hazard ratio was calculated using a Cox proportional hazard model with treatment as the only factor, and presented with its 95 % confidence interval from the Cox model and the 2-sided *P*-value from a log-rank test. Other time-to-event outcomes were analysed in the same way. For the primary statistical analysis events after more than 180 days (30 days for stroke) were considered censored. Additional analyses were performed for 180 days stroke, and using all available follow-up. All analyses were conducted with SAS v.9.3 (SAS Institute Inc., Cary, NC, USA). A two-tailed *P*-value <0.05 was considered statistically significant.

## Results

### Study population

Follow up was available for all patients up to 30 and 180 days as well as at the maximum follow up time, (median 25 months). Baseline, and procedural characteristics for all randomised patients have previously been published [1, 8].

### Clinical outcome

**Fig. 1:**
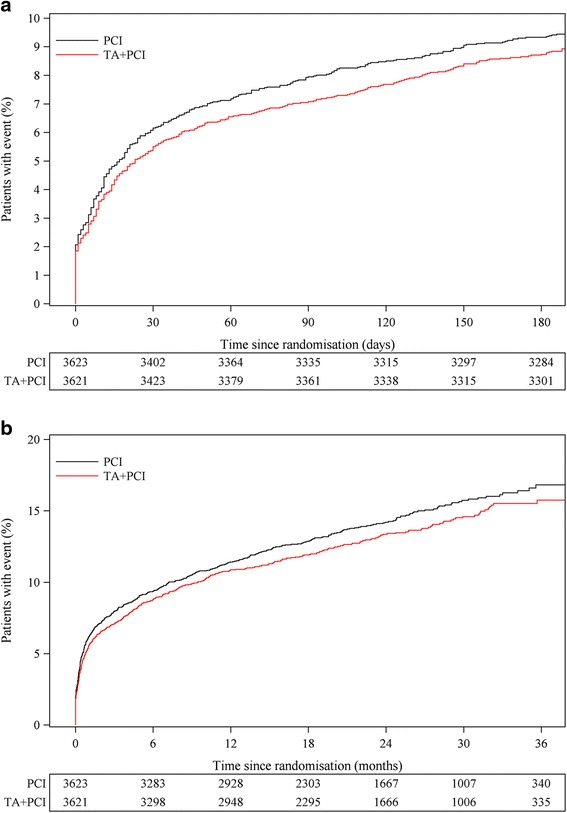
Time to event curves for the primary efficacy quadruple composite endpoint of CV death, cardiogenic shock, new hospitalisation for MI, and new hospitalisation for HF up to 180 days and to maximal follow up. Illustration of the Kaplan-Meier event curves depicting the cumulative probability of the composite endpoint of cardiovascular (CV) death, rehospitalisation for new myocardial infarct (MI), cardiogenic shock or new hospitalisation for heart failure (HF) up to 180 days (Panel **a**) (Hazard Ratio (HR) 0.93, 95 % CI (0.80-1.09), *P* = 0.36) or at maximal follow up (mean 25 months) (HR 0.92, 95 % CI (0.82-1.05), *P* = 0.21) (Panel **b**) following percutaneous coronary intervention (PCI) with thrombus aspiration (TA) vs. PCI alone

All analysed endpoints are listed in Table 1. For efficacy up to 180 days, there were no significant differences in outcome for the individual endpoints of all cause death, CV death, rehospitalisation with new MI, definite ST, and TVR or in combination with cardiogenic shock or new hospitalisation with HF. The primary endpoint consisting of the quadruple composite endpoint of CV death, cardiogenic shock, rehospitalisation with new MI, new hospitalisation for HF up to 180 days occurred in 8.7 % (316 of 3621) in the thrombus aspiration group compared to 9.3 % (338 of 3623) in the PCI alone group (hazard ratio (HR), 0.93; 95 % confidence interval (CI), 0.80 - 1.09; *P* = 0.36) (Fig. 1a), and at maximal follow up (25 months), in 13.3 % (483) vs. 14.4 % (521) (HR, 0.92; 95 % CI, 0.82-1.05; *P* = 0.21) (Fig. 1b).

**Table 1:** Outcomes for efficacy, net-benefit and safety up to 30 days, 180 days and to maximal follow-up (mean 25 months)

Outcome	Thrombus aspiration *N* = 3621	PCI alone *N* = 3623	Hazard ratio (95 % CI)	*P*-value
Efficacy up to 180 days				
Cardiovascular (CV) death	125 (3.5 %)	133 (3.7 %)	0.94 (0.74-1.20)	0.62
All cause death	145 (4.0 %)	156 (4.3 %)	0.93 (0.74-1.17)	0.52
Rehospitalisation for new myocardial infarction (Rehosp-MI)	63 (1.7 %)	70 (1.9 %)	0.90 (0.64-1.26)	0.53
Definite stent thrombosis (ST)	20 (0.6 %)	26 (0.7 %)	0.77 (0.42-1.37)	0.37
Target vessel revascularization (TVR)	130 (3.6 %)	144 (4.0 %)	0.90 (0.71-1.14)	0.38
Cardiogenic chock or new hospitalisation with heart failure (HF)	176 (4.9 %)	186 (5.1 %)	0.95 (0.77-1.16)	0.59
CV death, cardiogenic shock, Rehosp-MI or new heart failure (HF)^a^	316 (8.7 %)	338 (9.3 %)	0.93 (0.80-1.09)	0.36
Cardiovascular death, cardiogenic shock, Rehosp-MI, new HF, definite ST or TVR	409 (11.3 %)	448 (12.4 %)	0.91 (0.79-1.04)	0.15
Efficacy during follow-up (median 25 months)				
Cardiovascular death	215 (5.9 %)	229 (6.3 %)	0.94 (0.78 - 1.13)	0.51
Cardiovascular death, cardiogenic shock, Rehosp-MI or new HF^a^	483 (13.3 %)	521 (14.4 %)	0.92 (0.82-1.05)	0.21
Cardiovascular death, cardiogenic shock, Rehosp-MI, new HF, definite ST or TVR	615 (17.0 %)	678 (18.7 %)	0.90 (0.81-1.00)	0.058
Safety				
Stroke up to 30 days	24 (0.7 %)	27 (0.7 %)	0.89 (0.51-1.54)	0.68
Stroke up to 180 days	42 (1.2 %)	45 (1.2 %)	0.93 (0.61-1.42)	0.75
Stroke until follow up (median 25 months)	89 (2.5 %)	89 (2.5 %)	1.00 (0.75-1.34)	0.99
Net-benefit up to 180 days				
Cardiovascular death, cardiogenic shock, Rehosp-MI, new HF, or stroke^b^	343 (9.5 %)	371 (10.2 %)	0.92 (079-1.07)	0.27
Cardiovascular death, cardiogenic shock, Rehosp-MI, new HF, stroke, definite ST or TVR^c^	436 (12.0 %)	479 (13.2 %)	0.90 (0.79-1.03)	0.12
Net-benefit during follow-up (median 25 months)				
Cardiovascular death, cardiogenic shock, Rehosp-MI, new HF, or stroke^b^	545 (15.1 %)	582 (16.1 %)	0.93 (0.83-1.05)	0.24
Cardiovascular death, cardiogenic shock, Rehosp-MI, new HF, stroke, definite ST or TVR^c^	677 (18.7 %)	736 (20.3 %)	0.91 (0.82-1.01)	0.08

^a^Denotes the primary quadruple composite endpoint within 180 days and also the same quadruple endpoint, during follow up (median 25 months)

^b^Denotes the net-benefit composite endpoint up to 180 days and during follow-up

^c^Denotes the extended net-benefit composite endpoint within 180 days and during follow-up

**Fig. 2:**
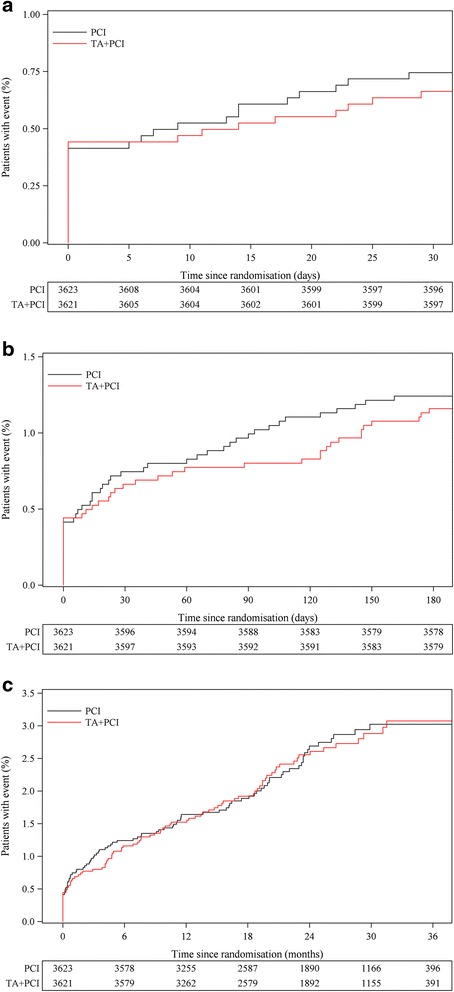
Time to event curves for the risk of stroke up to 30 and 180 days and to maximal follow up. Kaplan-Meier event curves showing cumulative probability of the endpoint of stroke up to 30 days (Hazard Ratio (HR) 0.89, 95 % CI (0.51-1.54), *P* = 0.68) (Panel **a**), 180 days (HR 0.93, 95 % CI (0.61-1.42), *P* = 0.75) (Panel **b**) and at maximal follow up (mean 25 months) (HR 1.00, 95 % CI (0.75-1.34), *P* = 0.99) (Panel **c**) following percutaneous coronary intervention (PCI) with thrombus aspiration (TA) vs. PCI alone

Stroke within 30 days occurred in 0.7 % (24) in the thrombus aspiration group vs. 0.7 % (27) of patients in the PCI alone group (HR, 0.89; 95 % CI, 0.51–1.54; *P* = 0.68) and within 180 days in 1.2 % (42) vs. 1.2 % (45), (HR, 0.93; 95 % CI, 0.61-1.42; *P* = 0.75) (Fig. 2a and b).

**Fig. 3:**
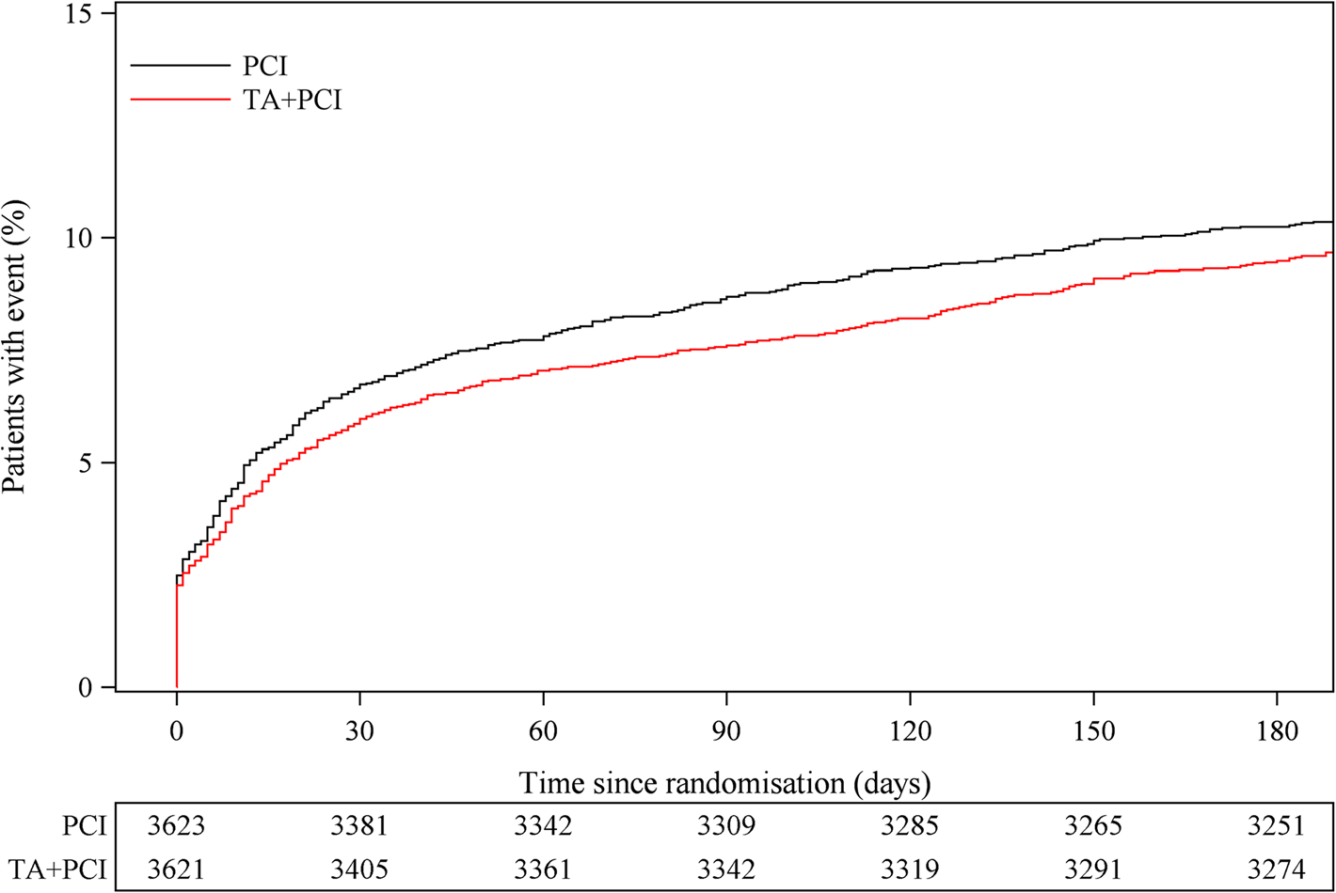
Time to event curve for the net-benefit composite endpoint up to 180 days. Kaplan-Meier event curves showing cumulative probability of the net-benefit outcome up to 180 days of cardiovascular (CV) death, development of cardiogenic shock, rehospitalisation for new myocardial infarction (MI), new hospitalisation for heart failure (HF) or stroke up to 180 days following percutaneous coronary intervention (PCI) with thrombus aspiration (TA) vs. PCI alone, Hazard Ratio 0.92, 95 % CI (079-1.07), *P* = 0.27

The net-benefit composite endpoint within 180 days, similar to the one analysed in TOTAL, of CV death, cardiogenic shock, rehospitalisation with new MI, new hospitalisation for HF or stroke occurred in 9.5 % (343) in the PCI plus thrombus aspiration (TA) group vs. 10.2 % (371) in the PCI alone group, (HR 0.92; 95 % CI, 0.79-1.07, *P* = 0.27) (Fig. 3).

**Fig. 4:**
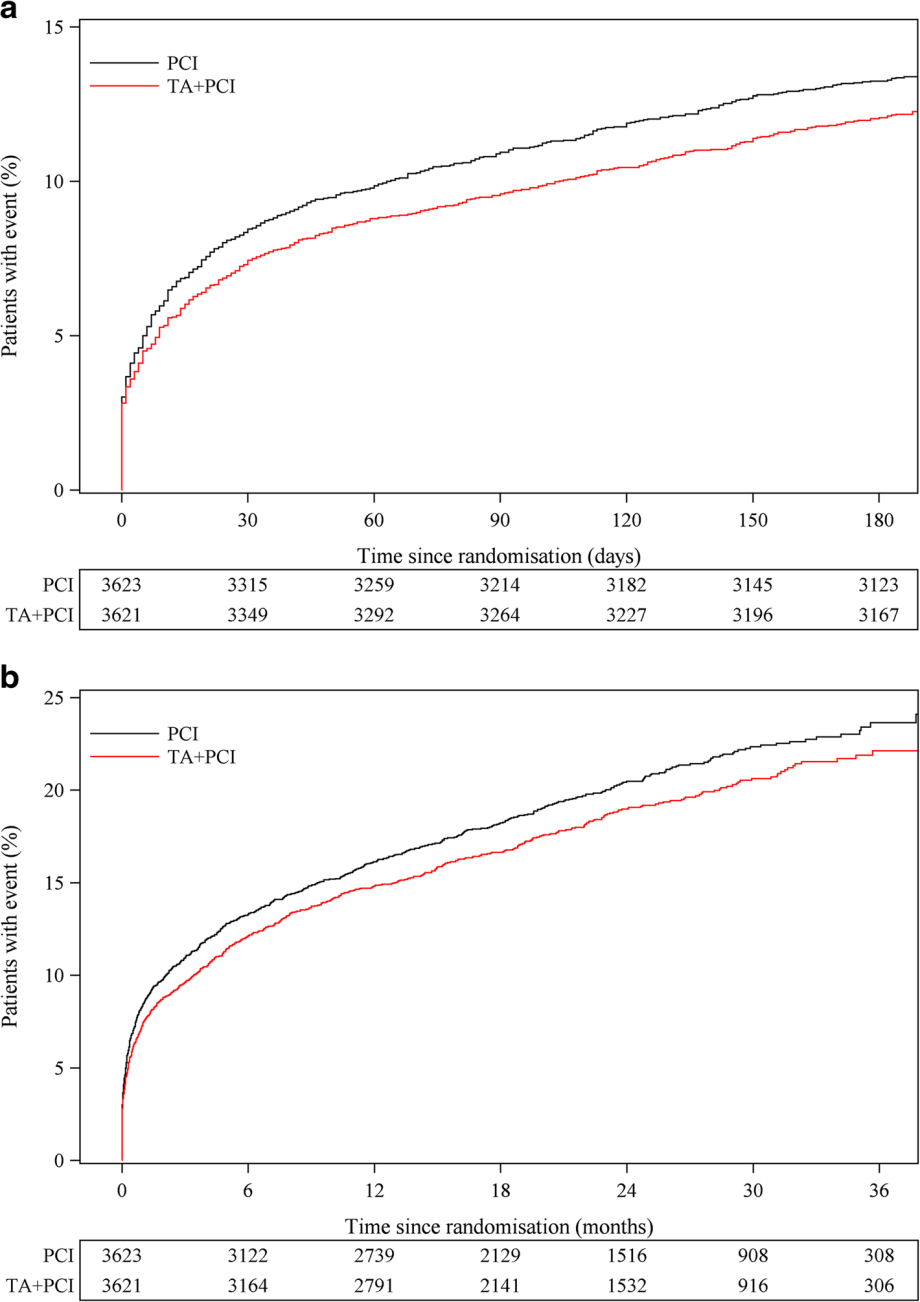
Time to event curves for the extended net-benefit composite endpoint up to 180 days and to maximal follow up. Illustration of the Kaplan-Meier event curves depicting the cumulative probability of the composite endpoint of cardiovascular (CV) death, cardiogenic shock, rehospitalisation for new myocardial infarct (MI), new hospitalisation for heart failure (HF), definite stent thrombosis (ST), target vessel revascularization (TVR), or stroke up to 180 days (Hazard Ratio (HR) 0.90, 95 % CI (0.79-1.03), *P* = 0.12) (Panel **a**) or at maximal follow up (HR 0.91, 95 % CI (0.82-1.01), *P* = 0.08) (mean 25 months) (Panel **b**), following percutaneous coronary intervention (PCI) with thrombus aspiration (TA) vs. PCI alone

The large extended net-benefit, composite endpoint of CV death, cardiogenic shock, rehospitalisation with new MI, new hospitalisation for HF, stroke, definite ST or TVR up to 180 days occurred in 12.0 % (436) vs. 13.2 % (479), (HR, 0.90; 95 % CI, 0.79 - 1.03, *P* = 0.12) (Fig. 4a), and at maximal follow up (25 months) in 18.7 % (677) vs. 20.3 % (736), (HR, 0.91; 95 % CI, 0.82-1.01, *P* = 0.08) (Fig. 4b) for patients treated with thrombus aspiration (TA) vs. PCI alone respectively.

## Discussion

In this post hoc analysis of the TASTE study comparing the composite endpoint of CV death, rehospitalisation with new MI, cardiogenic shock or new hospitalisation for HF up to 180 days between STEMI patients treated with thrombus aspiration and PCI vs. PCI alone there was no significant difference in outcome demonstrating a lack of clinical effect of thrombus aspiration. There was also no difference in stroke rates up to 30 or 180 days.

The allure of improving microvascular reperfusion in STEMI patients through thrombus aspiration is powerful, especially with the frequently observed removal of large macroscopic specimens. There are also indications that embolization of atherothrombotic materials may contribute to microvascular obstruction in humans [10]. Removal of thrombus would thus seem intuitive in order to improve outcome. However a host of thrombus aspiration trials have reported conflicting results and it was only following the single centre TAPAS trial that thrombus aspiration became widely accepted and upgraded in treatment guidelines [2, 11–13].

The TASTE trial was negative regarding the primary endpoint of death up to 30 days as well as all secondary endpoints including death at one year [1, 8]. Following the TAPAS and TASTE trials and several meta-analyses, there were conflicting data to support the use of routine thrombus aspiration as adjunctive treatment to PCI in STEMI patients [2, 13–15]. However, the TASTE and the TOTAL trials, which together randomised >17 000 patients, both demonstrated no clinical effect of routine use of thrombus aspiration in STEMI patients undergoing primary PCI. Both TASTE and TOTAL were similarly negative across the board in respect with all major secondary endpoints except stroke up to 30 days, which in TOTAL showed a small significantly increased risk of stroke in the thrombus aspiration group within 30 days. However, there were no demonstrated differences in stroke rates up to 30 or 180 days in the TASTE trial, similarly as reported in a meta-analysis by De Luca et al. [16]. Also, the excess of strokes in the thrombus aspiration group in TOTAL was exceedingly small (0.7 % vs. 0.3 %) with separating event curves after the stent procedure which may therefore be explained by a play of chance. With this study of the TASTE population using several composite endpoints and an extended composite endpoint, we were still unable to demonstrate any clinical effect of thrombus aspiration.

We can thus favourably compare the 7244 patient TASTE trial to the 10732 patient TOTAL trial and although no clinical follow up or adjudication of events were performed in TASTE, the outcomes are very similar across all endpoints, composite endpoints and subgroups except stroke. However, and most importantly, both primary endpoints conclude that there is no clinical benefit of routine thrombus aspiration during PCI in STEMI patients. In light of these results, the recently released AHA/ACC/SCAI focused update on primary PCI in STEMI patients has downgraded the use of thrombus aspiration in patients undergoing primary PCI for STEMI to Class III when used routinely and IIb for bailout use [17].

Because of the similar findings of the TASTE and TOTAL trial we now feel confident that with this simple type of registry based RCT (randomised clinical trial), the design of TASTE opens up a new venue of performing randomised clinical trials which can be undertaken at a fraction of the cost of a classical RCT, and can also be performed exceedingly quickly. Other advantages of basing randomised clinical trials on registries include comparison to the non-randomised cohort, and continuous follow up over time.

Thus, published randomised studies and registry studies as well as meta-analyses of randomised trials of thrombus aspiration have shown conflicting results with respect to mortality and other clinical outcomes [1–4, 14, 18, 19]. All these studies have their limitations, but in the light of the TASTE and TOTAL trials it is now established in our opinion that the *routine* use of thrombus aspiration during primary PCI in STEMI patients conveys no clinical benefit, as stated in the new guidelines [17].

Why does the current evidence demonstrate a lack of clinical effect of routine thrombus aspiration in STEMI patients? This could be explained simply through inefficient devices or wrong techniques for thrombus aspiration since recent optical coherence tomography (OCT) findings indicate large amounts of remaining thrombus following aspiration [20]. There is also the possibility that microvascular obstruction, which is well correlated to adverse outcomes, is multifactorial and more dependent on additional factors such as plaque/debris embolization, myocardial swelling, spasm or reperfusion injury [21, 22].

Does this mean that no patient should receive thrombus aspiration? This is currently unknown. Most interventional cardiologists who have aspirated large amounts of thrombus have difficulty imagining that some of these procedures were of no benefit to patients. Both the TASTE trial and TOTAL trial randomised patients to *routine* thrombus aspiration. Surprisingly there was no significant interaction for thrombus score and outcome of the randomised treatments in either TASTE or TOTAL, but bailout thrombus aspirations were nevertheless performed in 4.9 % and 7.1 % of patients. Thus, there may be a small number of patients in which thrombus aspiration cannot be completely discounted as beneficial in [23].

### Limitations

This is a post hoc retrospective analysis of the prospective R-RCT TASTE, using non-prespecified endpoints. As such, the validity of the data is lower than the prospective RCT TOTAL. All clinical events were solely gathered through existing national health registries and death registries with no adjudication of clinical events. Because all data were gathered from health and death registries without monitoring of individual patient files, we cannot be absolutely certain that there are no missing data or faulty registrations.

## Conclusions

This post hoc analysis of the TASTE study comparing the quadruple composite endpoint of CV death, rehospitalisation for new MI, cardiogenic shock and rehospitalisation for HF up to 180 days between STEMI patients treated with primary PCI with thrombus aspiration vs. PCI alone demonstrated no statistical difference in outcome or difference in stroke rates up to 30 or 180 days.

## Availability of data and materials

The data for the patients included in the TASTE study is stored at Uppsala Clinical Research Center, Dag Hammarskjölds Väg 50A, 752 37 Uppsala, Sweden. (http://www.ucr.uu.se/en).

## Abbreviations

CV: cardiovascular
HH: heart failure
HR: hazard ratio
ICD: International classification of disease
MI: myocardial infarction
NPR: The National Patient Registry
NYHA: New York Heart Association
PCI: percutaneous coronary intervention
RCT: randomized clinical trial
R-RCT: registry based randomised clinical trial
SCAAR: Swedish angiography and angioplasty registry
ST: stent thrombosis
STEMI: ST-elevation myocardial infarction
SWEDEHEART: Swedish Web-system for Enhancement and Development of Evidence-based care in Heart disease Evaluated According to Recommended Therapies registry
TASTE: The Thrombus Aspiration in ST-Elevation myocardial infarction in Scandinavia trial
TOTAL: A Randomised Trial of Routine Aspiration ThrOmbecTomy With PCI Versus PCI ALone in Patients With STEMI Undergoing Primary PCI
TVR: target vessel revascularization
